# Informed Consent and Surrogate Interference at the Initiation of Community-Based Palliative Care Services

**DOI:** 10.1089/pmr.2024.0018

**Published:** 2024-07-13

**Authors:** John C. Stys

**Affiliations:** Parkinson School of Health Sciences and Public Health, Loyola University Chicago, Chicago, Illinois, USA.

**Keywords:** autonomy, best interest, capacity, consent, duty of care, substituted judgment, surrogate

## Abstract

Community-based palliative care (CBPC) clinicians sometimes contend with an ethically charged scenario when they encounter patients for the first time: The patient’s spouse, or other loved one or caregiver, revokes the patient’s valid informed consent to initiate care. While surrogates are usually motivated by protective instincts, there are other situations where surrogates act out of self-interest. This article considers whether it is ever ethically justified for an adult to revoke another adult’s valid informed consent to initiate palliative care services. The article examines this scenario from three perspectives: the patient’s capacity to give or relinquish informed consent, the surrogate’s intent and use of substituted judgment or best interest, and the clinician’s duty to provide clinical care. This ethical analysis argues that CBPC clinicians have an ethical responsibility to provide palliative care services for patients who have given valid informed consent for those services even when a surrogate acts as an interfering or oppositional force.

## Introduction

Many Americans lack knowledge of palliative care’s existence and underappreciate its broad clinical value and juxtaposition to other clinical services. As recently as 2018, 71% of U.S. adults reported no knowledge of palliative care, and 48% misunderstood how palliative care differs from hospice care.^1,2^ At the turn of the millennium, hospice companies started adding “*and Palliative Care*” to their names. Many of these hospices envisioned census and revenue growth from an integrated referral funnel. Despite growing access to palliative care services, especially in the acute care setting,^2^ innovation and wide spread use of community-based palliative care (CBPC) services at scale remain cutting-edge while still associated with the hospice industry.^3^

Inadequate knowledge of palliative care, coupled with skepticism, creates conditions for distrust. It is common for potential consumers of palliative care services to conflate them with those offered by hospices.^4^ In doing so, it is understandable that some patients and families may have reservations. This can cause further confusion when CBPC clinicians arrive at the patient’s home to enroll them in services, and there is what one might call a “disagreement at the door.” That is, while many patients and their identified surrogate decision-makers agree about enrolling in new health care services, sometimes they do not. As clinicians, it is common to assume that a patient’s surrogate strives to act in the patient’s best interest. But some surrogates may act unethically by revoking the patients previously expressed, valid, and informed consent for services.^4^ This article considers a case in which one adult interferes with another’s decision to receive CBPC. First, it examines patient autonomy and the patient’s capacity to consent to services or relinquish that decision to someone else. Second, it examines the surrogate’s capacity, ability to act from substituted judgment or best interest, and beneficent or maleficent intent. The article suggests that CBPC clinicians have an ethical obligation to provide the treatment to which the patient consented, even when a surrogate acts as an interfering or oppositional force.

## Background

Although the structure, goals, and day-to-day practice of CBPC have matured over the past several decades, large-scale programmatic execution remains fertile ground for innovation.^5^ The introduction of this innovation has prompted certain companies in the palliative care sector to collaborate with payer organizations to screen communities for individuals who require care but have not yet received it.^6^ Upon identification of patients who would benefit from palliative care, the payor and provider teams utilize the information gathered from the screening process to reach out and attempt to enroll their seriously ill, covered members. The aim is to alleviate symptom burden and decrease the costs associated with hospitalizations. Despite engaging in initial discussions with the patient regarding CBPC through the screening, engagement, and scheduling processes, it is uncertain what the patient comprehends or anticipates from the palliative care team. During the telephone-based screening process, there are instances where the patient believes they have agreed to a future informational home visit, while others believe they have already provided consent for treatment. In certain cases, despite the patient’s response during the screening call, a surrogate intervenes by revoking the patient’s validly expressed consent to initiate palliative care services. This presents the clinician with a challenging decision: to advocate for the patient’s previously communicated choice or withdraw in the face of a surrogate who rejects services on the patient’s behalf.

## Case Study

A 74-year-old woman experiences pain and new functional limitations related to active malignancies. She shares an apartment with her husband of 48 years in an assisted living facility catering to an affluent demographic. Both individuals can communicate their health care needs and wishes. While the patient trusts her health care providers, her husband has grown skeptical of the health care system and believes older adults are targets for identity theft, financial schemes, and health care-related scams.

The patient’s insurance carrier includes CBPC as part of the patient’s plan. When the nurse practitioner arrives at the assisted living facility (ALF) to enroll the patient, she discusses the benefits of CBPC and presents the patient with a consent form. After the patient signs it, the surrogate emerges from another room. Interrupting the visit, he says, “We don’t want your services. Please leave.” Confused, the nurse practitioner, acting as the patient’s advocate, explains to the surrogate that the patient communicated her desire for the services and signed a consent form. The patient and the surrogate leave the room to have a brief conversation. When they reemerge, the patient tells the nurse practitioner that she will withdraw her informed consent and politely declines service initiation.

## The Patient: Capacity to Give or Relinquish Informed Consent

Ethically, the patient’s ability to exercise her autonomy is at stake. Autonomy is concerned with a person’s freedom to make a reasoned decision and act upon it independently without external influence.^7^ For the patient to give informed consent, she must have the capacity to understand potential risks and benefits and to consent voluntarily.^8^ The concept of capacity is related to one’s ability to act autonomously, but focusses “on whether such persons are capable, cognitively, psychologically, and legally, of adequate decision-making.”^9^

If the patient’s capacity to consent to services is deemed intact after the nurse practitioner’s initial assessment, the clinician may proceed with confidence. The patient’s choice to accept palliative care services is ethically appropriate. Sometimes, despite the patient having the capacity to make health care decisions, the patient might choose to allow the surrogate to participate in the decision-making process. Levinson, et al. found that individual preferences for exercising one’s autonomy varies significantly.^10^ However, a clinician may encounter a patient with the capacity to make health care decisions but watches as the surrogate attempts to revoke the patient’s informed consent based on a misconstrued understanding of substituted judgment. Substituted judgment occurs when the patient’s wishes are known and are meant to be followed as if the patient would have if able to do so. Because the wishes are known, the surrogate is bound to carry out the patient’s wishes as documented.^9^ When the surrogate acts such that their motives are questionable and colored by the surrogate’s self-interest, the clinician should pause and consider how to proceed. The clinician should advocate for the patient’s wishes while sensitively negotiating the growing risk for interpersonal conflict.

Alternatively, when the patient lacks the capacity to consent, the clinician should follow the state’s surrogacy act or other similar legislation. It is appropriate to address advance care planning with the surrogate. As someone with a legal or emotional stake in the patient’s well-being, the surrogate should act according to substituted judgment. When the patient’s wishes are unknown or not documented, the surrogate should act in the patient’s best interest. Acting in this way is grounded in the surrogate’s attempt to obtain the highest quality and most relevant clinical outcomes.^11^ The surrogate acting in this manner is the ethically appropriate way to proceed. However, if the clinician judges that the surrogate is making decisions that are not objectively in the patient’s best interest, the clinician has an ethical obligation to pause, reassess the scenario, and potentially call upon the interdisciplinary team for support.

## The Surrogate: Acting in Best Interest or Self Interest?

A patient with the capacity to make health care decisions might relinquish that power to a surrogate. The patient may want the surrogate to participate equally in the decision-making process.^12^ The patient may lack confidence in their ability to decide; the patient has capacity but is unsure how to proceed. The patient might lack confidence in the decision itself, doubting the quality of the choice. The surrogate may have a history of manipulating or even abusing the patient. In that case, the patient may relinquish decision-making power out of fear. The patient may believe that while they both can make independent decisions, the patient may believe that their wishes are subordinate to the surrogate’s. Browner describes how some patients feel obligated to please the surrogates on whom they depend.^11^ One way this obligation manifests is the patient passively allowing the surrogate to revoke their preferences or consent for care.^13^ Shafir believes choices generate conflict.^14^ The patient might know that making a self-directed choice will cause disharmony, and that the most efficient means to maintain peace and equilibrium in the relationship is to yield to the surrogate.

Clinicians who believe a surrogate is acting aggressively paternalistic witnesses an affront to the patient’s autonomy. If the surrogate acts in this way, it may be a sign of manipulation that benefits the partner’s interests. The clinician might observe different forms of the surrogate’s self-serving actions. The surrogate might exert an intimidating physical presence, hold emotional or financial power in the relationship, possess better health and functional ability, or possess a louder voice that demands attention. Those who believe the partner’s paternalistic act is ethical may base their opinion on a belief that no person’s autonomy is absolute. The duty to protect autonomy can conflict with a responsibility to promote other goods.^11^ The surrogate’s seemingly overbearing attitude may signal an attempt to preserve the patient’s best interest—in this case, preventing exposure to a scam. The surrogate may possess more sophisticated medical knowledge or even possess valuable new information not yet communicated to the patient.^15^ If the patient understands these characteristics as the surrogate’s qualities, the patient may be willing to relinquish their informed consent. Wilson et al. advocate for a broader, even communal, understanding of autonomy. They suggest that “An individualized approach to autonomy fails to recognize the complexity of decision-making including the cultural and social relations that facilitate patient involvement in decision-making.”^15^

## Conclusion

This analysis of patient-informed consent and surrogate interference highlights common scenarios and ethical concepts a CBPC clinician may face in their practice. The concepts include autonomy, capacity, beneficent and maleficent intent, and paternalistic nudging. Scenarios related to the themes raised in this article will always be unique to the time, place, clinical acuity, and people present. As such, this article does not attempt to provide ethically actionable interventions appropriate to all scenarios.

There are three steps that a clinician may take that could help to lower the ethical burden upon meeting the patient and surrogate. First, consider whether the patient’s capacity requires a formal evaluation when first introduced. Second, pause for a moment. Rushton highlights the urgency sometimes experienced when clinicians face an ethical conflict. She suggests the pause creates space for “everyone involved in a conflict situation to mindfully consider the right and good thing to do.”^16^ Third, observe and evaluate the surrogate for motives appearing unusual or incongruous with the patient’s narrative. As outlined in Provision 2 of the American Nurses Association’s Code of Ethics, the nurse clinician’s primary obligation is to the patient while, at the same time, considering the patient within the broader context of family and community.^16^

The clinician cannot ignore the obligation to advocate for the patient when a surrogate attempts to reject, revoke, or otherwise dominate the clinician–patient relationship. This is especially true in the community setting when the clinician practices alone. Without immediately accessible colleagues, the palliative care clinician must depend on their internal code of ethics paired with intuition to protect the patient’s interests. In most cases, it is likely the surrogate misunderstands the reason for the home visit or even how the clinician conducts health care services in a residential setting. In that case, the clinician’s nudging could be the remedy to a scenario that might otherwise turn into conflict resulting in a patient going without the care they need. In any of these scenarios, the clinician should never abandon the patient after a less-than-perfect initial interaction.

## Abbreviation Used

ALF: Assisted living facility
